# Effects of Incubation Time and Inoculation Level on the Stabilities of Bacteriostatic and Bactericidal Antibiotics against *Salmonella* Typhimurium

**DOI:** 10.3390/antibiotics10081019

**Published:** 2021-08-22

**Authors:** Nana Nguefang Laure, Jirapat Dawan, Juhee Ahn

**Affiliations:** 1 Department of Biomedical Science, Kangwon National University, Chuncheon 24341, Gangwon, Korea; 202016039@kangwon.ac.kr; 2 Institute of Bioscience and Biotechnology, Kangwon National University, Chuncheon 24341, Gangwon, Korea

**Keywords:** antibiotic susceptibility, antibiotic stability, bacteriostatic antibiotic, bactericidal antibiotic, inoculum effect

## Abstract

This study was designed to evaluate the stability of chloramphenicol, erythromycin, tetracycline, cephalothin, ciprofloxacin, and tobramycin against antibiotic-sensitive *Salmonella* Typhimurium (ASST) and antibiotic-resistant *S*. Typhimurium (ARST) during the broth microdilution assay. The antimicrobial activity in association with antibiotic stability was measured by using antibiotic susceptibility, time-delayed inoculation, time-extended incubation, and inoculum effect assays. The loss of the antimicrobial activity of cephalothin against ASST exposed to 1 MIC was observed for the 10 h delayed inoculation. The antimicrobial activities of tetracycline and ciprofloxacin against ASST and ARST exposed to ½ MIC were significantly decreased after the 10 h delayed inoculation. All antibiotics used in this study, except for ciprofloxacin, showed the considerable losses of antimicrobial activities against ASST and ARST after 40 h of incubation at 37 °C when compared to the 20 h of incubation during AST. Compared to the standard inoculum level (6 log CFU/mL), the MIC_0.1_ values of bactericidal antibiotics, ciprofloxacin and tobramycin against ASST were increased by more than 4-fold at the high inoculum level of 9 log CFU/mL. This would provide practical information for better understanding the clinical efficacy of the currently used antibiotics by considering the antibiotic stability during incubation time at different inoculum levels.

## 1. Introduction

Antibiotics are mainly classified based on their target sites, including the inhibition of cell wall synthesis, protein synthesis, nucleic acid synthesis, membrane function, and metabolic pathway [1,2]. These antibiotics act through different mechanisms of action against bacteria [2]; bacteriostatic antibiotics include clindamycin, chloramphenicol, erythromycin, tetracycline, and trimethoprim, whereas bactericidal antibiotics include gentamicin, kanamycin, ciprofloxacin, and tobramycin [3,4]. Antibiotic susceptibility testing (AST) is widely used to select appropriate and effective treatment options that play an important role in making clinical decision to treat bacterial infections [5,6,7]. Although the antibiotic potential is commonly evaluated by using gold standard methods such as disc diffusion and broth microdilution, the quantitative and qualitative AST results do not provide sufficient information on the mechanisms of antibiotic action [6,8,9]. Furthermore, the modes of action of antibiotics against bacteria vary with growth medium, inoculum level, and incubation period [3,10,11]. Therefore, it is necessary to re-evaluate the in vitro antibiotic stability under different test conditions.

The accurate determination of the antibiotic susceptibility of bacteria remains a key factor for optimizing the antibiotic treatment regimen in association with pharmacokinetic (PK) and pharmacodynamic (PD) properties [12]. However, during the AST, the degradation of antibiotics may be attributed to the growth condition, bacterial inoculum, incubation time, temperature, and growth medium [3,5,11]. The evaluation of the clinical efficacy of antibiotics needs to take into consideration the antibiotic degradation [12]. The overestimation or underestimation of antibiotic efficacy leads to the misprescription of antibiotics and the accumulation of residual antibiotics, contributing to antibiotic resistance in bacteria [11,13]. However, little attention has been paid to the antibiotic stability during the AST. Therefore, the objective of this study was to assess the stability of bacteriostatic (chloramphenicol, erythromycin, and tetracycline) and bactericidal (cephalothin, ciprofloxacin, tobramycin) antibiotics against *Salmonella* Typhimurium under the conditions of time-delayed inoculation, time-extended incubation, and different inoculum levels.

## 2. Results

### 2.1. Stability of Antibiotics in the Media

**Figure 1:**
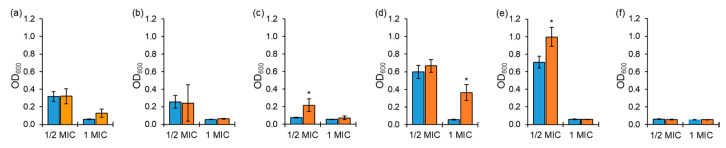
Growths of antibiotic-sensitive *Salmonella* Typhimurium ATCC 19585 (ASST) exposed to ½ and 1 MICs of chloramphenicol (**a**), erythromycin (**b**), tetracycline (**c**), cephalothin (**d**), ciprofloxacin (**e**), tobramycin (**f**) at 0 h (■) and 10 h (■) delayed inoculation. * indicates the significant difference within MIC at *p* < 0.05.

The antibiotic susceptibility testing against ASST and ARST was conducted to evaluate the antibiotic stability in media at the 0 h and 10 h delayed inoculation (Figure 1). Cephalothin and ciprofloxacin showed a significant loss in activity against ASST exposed to ½ MIC at the 0 h inoculation compared to 1 MIC (Figure 1d,e). No significant difference in the antimicrobial activity of tobramycin was observed against ASST between exposures to ½ MIC and 1 MIC (Figure 1f). The cephalothin activity against ASST exposed to 1 MIC was decreased at the 10 h delayed inoculation compared to the 0 h inoculation (Figure 1d). The loss of tetracycline and ciprofloxacin activity against ASST exposed to ½ MIC was observed for the 10 h delayed inoculation compared to the 0 h inoculation (Figure 1c,e).

**Figure 2:**
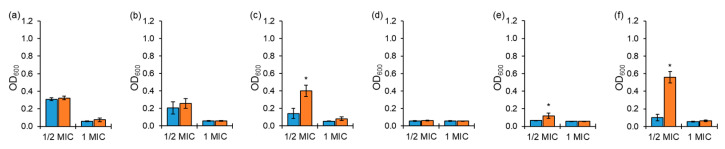
Growths of antibiotic-resistant *Salmonella* Typhimurium CCARM 8009 (ARST) exposed to ½ and 1 MICs of chloramphenicol (**a**), erythromycin (**b**), tetracycline (**c**), cephalothin (**d**), ciprofloxacin (**e**), tobramycin (**f**) at 0 h (■) and 10 h (■) delayed inoculation. * indicates the significant difference within MIC at *p* < 0.05.

The antimicrobial activities of chloramphenicol and erythromycin against ARST exposed to ½ MIC were significantly decreased at the 0 h inoculation after 20 h of incubation at 37 °C compared to 1 MIC (Figure 2a,b), while those of tetracycline, cephalothin, ciprofloxacin, and tobramycin against ARST showed no significant difference between exposures to ½ MIC and 1 MIC at the 0 h inoculation (Figure 2c,f). No significant losses in antimicrobial activities against ARST exposed to 1 MIC of all antibiotics were observed between the 0 h inoculation and the 10 h delayed inoculation, while the antimicrobial activities of tetracycline, ciprofloxacin, and tobramycin against ARST exposed to ½ MIC were significantly lost at the 10 h delayed inoculation compared to the 0 h inoculation (Figure 2c,e,f).

### 2.2. Sustainability of Antimicrobial Activity

**Figure 3:**
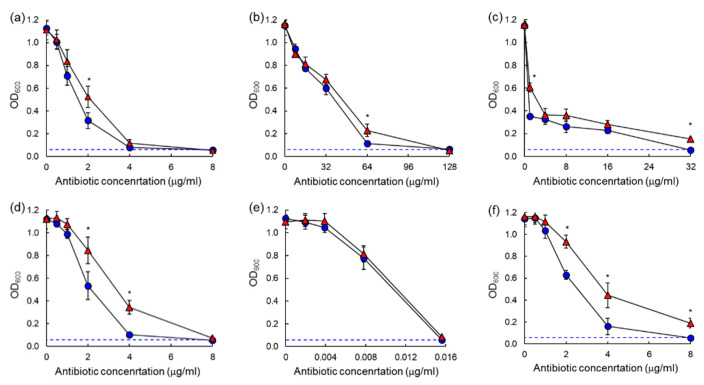
Dose–response curves of antibiotic-resistant *Salmonella* Typhimurium ATCC 19585 (ASST) exposed to different concentrations of chloramphenicol (**a**), erythromycin (**b**), tetracycline (**c**), cephalothin (**d**), ciprofloxacin (**e**), tobramycin (**f**) after 20 h (●) and 40 h (▲) of incubation. * indicates the significant difference within MIC at *p* < 0.05.

**Figure 4:**
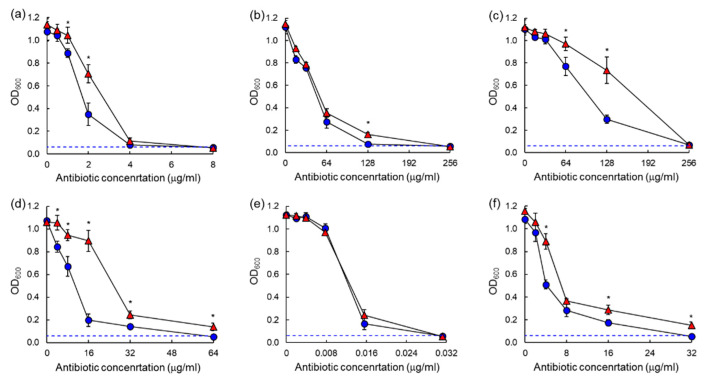
Dose–response curves of antibiotic-sensitive *Salmonella* Typhimurium CCARM 8009 (ARST) exposed to different concentrations of chloramphenicol (**a**), erythromycin (**b**), tetracycline (**c**), cephalothin (**d**), ciprofloxacin (**e**), tobramycin (**f**) after 20 h (●) and 40 h (▲) of incubation. * indicates the significant difference within MIC at *p* < 0.05.

The antibiotic susceptibilities against ASST and ARST were evaluated after 20 h and 40 h of incubation as shown in the dose–response curves (Figure 3 and Figure 4). All antibiotics used in this study showed significant losses of antimicrobial activity against both ASST (Figure 3) and ARST (Figure 4) after 40 h of incubation with the exception of ciprofloxacin. The MIC values of tetracycline (>32 µg/mL) and tobramycin (>8 µg/mL) against ASST (Figure 3c,f), and those of cephalothin (>64 µg/mL) and tobramycin (>32 µg/mL) against ARST (Figure 4d,f), were increased after 40 h of incubation at 37 °C.

### 2.3. Inoculum Effect

**Figure 5:**
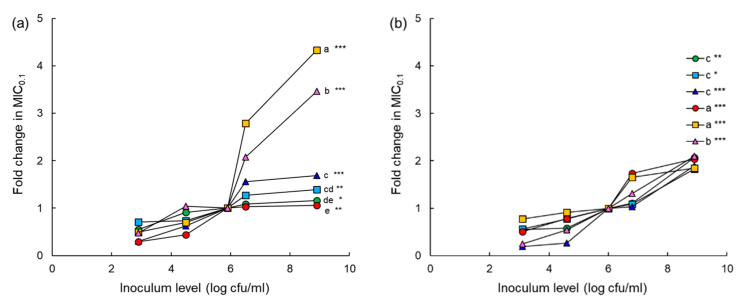
Inoculum effect of chloramphenicol (CHL; ●), erythromycin (ERY; ■), tetracycline (TET; ▲), cephalothin (CEP; ●), ciprofloxacin (CIP; ■), tobramycin (TOB; ▲) against different levels of antibiotic-sensitive *Salmonella* Typhimurium ATCC 19585 (ASST; (**a**)) antibiotic-resistant *S*. Typhimurium CCARM 8009 (ARST; (**b**)). Markers with different letters within an inoculum level (a–e) are significantly different among antibiotics at *p* < 0.05. *, **, and *** indicate significant difference between high (>6 log CFU/mL) and low (<5 log CFU/mL) inoculum levels at *p* < 0.05, *p* < 0.01, and *p* < 0.001, respectively.

The fold changes in the MIC_0.1_ values of chloramphenicol, erythromycin, tetracycline, cephalothin, ciprofloxacin, and tobramycin were compared to evaluate the inoculum effect (Figure 5). The high inoculum levels (6.5 and 9 log CFU/mL) showed a noticeable increase in the MIC_0.1_ values of all antibiotics against both ASST and ARST compared to the low inoculum levels (4.5 and 5 log CFU/mL). The highest fold change in the MIC_0.1_ of ciprofloxacin was observed at the inoculum of 9 log CFU/mL of ASST, showing a more than 4-fold increase, followed by tobramycin (>3-fold) (Figure 5a). The MIC_0.1_ values of all antibiotics against ARST were increased up to 2-fold at the inoculum of 9 log CFU/mL (Figure 5b).

## 3. Discussion

The antimicrobial activity is directly associated with the antibiotic stability during the AST. For instance, the incubation and inoculation conditions can affect the degradation of antibiotics during the broth microdilution assay, ultimately resulting in inaccurate AST results. Therefore, the AST results might not provide sufficient information on the antibiotic stability over the incubation period [14]. Cephalothin and ciprofloxacin showed the concentration-dependent activity against ASST (Figure 1). The concentration-dependent antibiotics inhibit bacterial growth proportionally with increasing concentrations of antibiotics, such as aminoglycosides and fluoroquinolones, while the time-dependent antibiotics increase the activity against bacteria up to MICs of antibiotics such as β-lactams and oxazolidinones [3]. The significant losses in tetracycline and cephalothin activities were observed at ½ MIC and 1 MIC, respectively, against ASST (Figure 1c,d). These antibiotics were unstable in aqueous solution, leading to a reduced half-life [15,16]. As shown in Figure 2, tetracycline, ciprofloxacin, and tobramycin were unstable to media at ½ MIC against ARST. Factors influencing the stability of antibiotics include light, media, pH, and temperature [11,17]. These losses of antimicrobial activity at the 10 h delayed inoculation indicates that the antibiotics were susceptible to the exposed conditions, such as media and incubation temperature [18]. This is in good agreement with the previous result that β-lactam antibiotics were degraded during the AST [5]. The growth media containing metals contributed to the degradation of the β-lactam antibiotics [5]. In addition, a previous study reported that the stability of tetracycline in fresh media was reduced after incubation in aged media. The loss in antimicrobial activities was due to the dissolved oxygen during incubation [19]. Therefore, the instability of antibiotics may cause sublethal effects, resulting in the development of antibiotic resistance in bacteria [20,21,22]. However, chloramphenicol, erythromycin, and tobramycin were stable to retain the antimicrobial activity against ASST exposed to both ½ MIC and 1 MIC during the AST (Figure 1), and the antimicrobial activities of chloramphenicol, erythromycin, and cephalothin remained unchanged during the AST (Figure 2). The results suggest that the stability of antibiotics under AST depends on the classes of antibiotics, concentrations of antibiotics, and degree of antibiotic resistance.

The dose–response curves for chloramphenicol, erythromycin, tetracycline, cephalothin, and tobramycin showed a significant decrease in antimicrobial activity against both ASST and ARST after 40 h of incubation when compared to 20 h (Figure 3 and Figure 4). The results suggest that ASST and ARST developed the ability to survive prolonged periods of exposure to antibiotics. This observation is in good agreement with the previous report that the development of resistance was noticeable when bacteria were exposed to sublethal concentrations of antibiotics [23]. The MIC values of tetracycline and tobramycin were increased against ASST after an extended incubation time of 40 h, while those of cephalothin and tobramycin were increased against ARST (Figure 3 and Figure 4). On the other side, these results indicate the loss of antibiotic activity throughout the incubation period [11]. This observation is in good agreement with the previous report that the antibiotic concentration was decreased over long-term incubation, leading to the increase in MIC [24]. A similar dose–response curve of ciprofloxacin against ASST and ARST was observed after 20 h and 40 h of incubation. This confirms that ciprofloxacin was relatively stable when compared to other classes of antibiotics [25]. The degradation of the antibiotics result from nutrient media, temperature, and test bacteria during the incubation period can cause a misreading of AST results [26]. This suggests that proper interpretations of the MIC results are essential to estimate the clinical efficacy of antibiotics. Therefore, the underestimation of antibiotic activity can cause substantial economic loss and public health risk [11].

The antimicrobial activity varies with the classes of antibiotics [10]. Tetracycline reversibly targeting the 30S ribosomal subunit can inhibit the binding of aminoacyl tRNA to the ribosome [27]. Chloramphenicol targeting the 50S ribosomal subunit can prevent the formation of peptides. In contrast, the ribosome-targeting aminoglycosides, such as streptomycin and kanamycin, can irreversibly bind to the 30S ribosomal subunit, leading to the inhibition of initiation and the induction of mistranslation [27]. The reversible antibiotics are effective against fast-growing bacteria, whereas the irreversible antibiotics are effective against slow-growing bacteria [28]. Bacteriostatic antibiotics are effective against bacterial persister cells by inhibiting protein synthesis [3]. The terms, bactericidal and bacteriostatic antibiotics, can be defined at the in vitro test, depending on the antibiotic classes and test strains [3]. The bacteriostatic antibiotics exhibit bactericidal activity at high concentrations, while the bactericidal antibiotics show bacteriostatic activity at low concentrations [3].

The susceptibilities of ASST and ARST to chloramphenicol, erythromycin, tetracycline, cephalothin, ciprofloxacin, and tobramycin varied among inoculum levels (Figure 5). The fold changes in the MIC_0.1_ values of all antibiotics tested in this study were significantly increased as the inoculum levels of ASST and ARST increased, known as inoculum effect [29]. This is in good agreement with previous studies that bacteria showed antibiotic susceptibility at the standard inoculums (10^5^ to 10^6^ CFU/mL) but antibiotic resistance at high inoculum levels [28,29]. The inoculum effect is responsible for the reduction in antimicrobial activity and the enhanced antibiotic resistance [30]. Accordingly, the inoculum effect is a major consideration to evaluate the antimicrobial activity during the AST [31]. The inoculum effect of ciprofloxacin was considerably increased against ASST, showing more than 4-fold change in MIC_0.1_ value (Figure 5a). This observation might be due to the active efflux pump that can be specific for ciprofloxacin as substrate or the relatively low MIC_0.1_ values against ASST. This is in good agreement with the efflux-mediated resistance to fluoroquinolones in many bacterial populations [29,32]. Cephalothin showed a comparably high inoculum effect against ARST (Figure 5b). The result implies that the inoculum level of ARST was less susceptible to β-lactam antibiotics because of the elevated production of β-lactamases [31]. The emergence of resistant mutants is more likely to be increased within large bacterial populations [31]. Therefore, the antibiotic susceptibility of bacteria depends on the level of bacterial load and the degree of antibiotic resistance [33,34].

## 4. Materials and Methods

### 4.1. Bacterial Strains and Culture Conditions

Strains of antibiotic-sensitive *Salmonella* Typhimurium ATCC 19585 (ASST) and antibiotic-resistant *S. Typhimurium* CCARM 8009 (ARST) were obtained from American Type Culture Collection (ATCC, Manassas, VA, USA) and Culture Collection of Antibiotic Resistant Microbes (CCARM, Seoul, Korea), respectively. The strains were sub-cultured at 37 °C for 20 h in trypticase soy broth (TSB) (BD, Becton, Dickinson and Co., Sparks, MD, USA). The activated cells were collected at the late exponential phase by centrifugation at 5000× *g* for 10 min at 4 °C. The harvested cells were washed twice with phosphate-buffered saline (PBS, pH 7.2) and adjusted to 10^8^ CFU/mL.

### 4.2. Antibiotic Susceptibility Assay

The susceptibility of ASST and ARST to chloramphenicol, erythromycin, tetracycline, cephalothin, ciprofloxacin, and tobramycin (Table 1) was evaluated by broth microdilution assay [35]. The antibiotic stock solutions were prepared at a final concentration of 1024 mg/mL by dissolving in ethanol (chloramphenicol, erythromycin, and tetracycline), water (cephalothin and tobramycin), and acetic acid (ciprofloxacin). Each antibiotic stock was serially (1:2) diluted ranging from 1024 µg/mL with TSB in 96-well microtiter plates (BD Falcon, San Jose, CA, USA) and inoculated with 10^6^ CFU/mL of ASST and ARST. The plates were incubated for 20 h at 37 °C to determine the minimum inhibitory concentration (MIC) of each antibiotic.

### 4.3. Time-Delayed Inoculation Assay

The antibiotic stability was evaluated in bacterial culture media by using a delay-time assay [5]. ASST or ARST was inoculated at the level of 10^6^ CFU/mL in 0 h incubated and 10 h delay incubated 96-well microtiter plates containing ½ MIC and 1 MIC of antibiotics. The growth of each test strain was measured after 20 h of incubation at 600 nm using a microplate reader (BioTek Instruments, Inc., Norwood, MA, USA).

### 4.4. Time-Extended Incubation Assay

The degradation of the antibiotics used in this study was evaluated by broth microdilution assay [35] with a slight modification. Each antibiotic stock was serially (1:2) diluted from 1024 µg/mL with TSB in 96-well microtiter plates (BD Falcon, San Jose, CA, USA), and ASST and ARST were inoculated at 10^5^ CFU/mL. After 20 h and 40 h of incubation at 37 °C, the dose–response curves of ASST and ARST were generated to evaluate the changes in antibiotic susceptibility.

### 4.5. Estimation of Inoculum Effect

The inoculum effect on antibiotic activity was evaluated by comparing MICs determined at different inoculum levels of ASST and ARST [31]. The test strains were diluted with fresh TSB to obtain different inoculum levels ranging from 8.2 × 10^2^ to 8.2 × 10^9^ CFU/mL and inoculated in each well of 96-well microtiter plates containing antibiotics serially (1:2) diluted from 1024 to 0 µg/mL. MIC_0.1_ values were determined at the lowest concentrations of antibiotics at which the optical density (OD) at 600 nm reached 0.1 after 20 h of incubation at 37 °C. The fold change was determined by the ratio of the MIC_0.1_ of each antibiotic at different inoculum levels to the standard inoculum level (10^6^ CFU/mL).

### 4.6. Statistical Analysis

The experiments were carried out in duplicate for three replicates. All data were analyzed by general linear model (GLM) and Fisher’s least significant difference (LSD) to determine significant differences at 5%, 1%, and 0.1% significance levels. The nonlinear curve fitting function of Microcal Origin^®^ (Microcal Software Inc., Northampton, MA, USA) was used to determine the MIC_0.1_ values of the antibiotics.

## 5. Conclusions

This study describes the effects of incubation time and inoculum level on antibiotic stability. AST is the first step to evaluate the antimicrobial potential and then determine the effective antibiotic treatment of bacterial infection. The most significant findings in this study were that the stabilities of chloramphenicol, erythromycin, tetracycline, cephalothin, ciprofloxacin, and tobramycin during the AST were highly influenced by incubation time, which might be a key factor in determining antimicrobial sustainability and antibiotic concentration; in addition, the antibiotic susceptibility of ASST and ARST was decreased at a high inoculum level when compared to standard inoculum, showing the noticeable inoculum effect of ciprofloxacin against ASST and cephalothin against ARST. Antibiotic stability is considered as an important factor for successful chemotherapeutic use. Thus, the AST might not provide sufficient information about the efficacy of antibiotics in association with the antibiotic stability under the incubation and inoculation conditions. The misinterpretation of the results obtained from the AST ultimately results in the underestimation or overestimation of antibiotic efficacy in clinical practice. Therefore, the current antibiotic susceptibility assays need to be re-evaluated by taking all test conditions (incubation time and inoculation level) into account in providing clinical guidelines and recommendations for chemotherapy and accurately predicting pharmacokinetics and pharmacodynamics in vivo.

## Figures and Tables

**Table 1:** Characteristics of antibiotics used in this study.

Antibiotic	Abbreviation	Class	Target Site	Polarity	Spectrum	MIC (μg/mL)	
						ASST	ARST
Chloramphenicol	CHL	Amphenicol	Peptidyl transferase	Hydrophobic	Bacteriostatic	4	4
Erythromycin	ERY	Macrolide	50S ribosome	Hydrophobic	Bacteriostatic	128	128
Tetracycline	TET	Tetracyclines	30S ribosome	Hydrophobic	Bacteriostatic	32	256
Cephalothin	CEP	β-lactam	Cell wall	Hydrophilic	Bactericidal	8	64
Ciprofloxacin	CIP	Fluoroquinolone	DNA gyrase	Hydrophobic	Bactericidal	0.0156	0.0312
Tobramycin	TOB	Aminoglycoside	30S ribosome	Hydrophilic	Bactericidal	8	32

## Data Availability

Not applicable.
